# Parental divorce’s long shadow: Elevated stroke risk among older Americans

**DOI:** 10.1371/journal.pone.0316580

**Published:** 2025-01-22

**Authors:** Mary Kate Schilke, Philip Baiden, Esme Fuller-Thomson

**Affiliations:** 1 Department of Psychology, Tyndale University, North York, Ontario, Canada; 2 School of Social Work, The University of Texas at Arlington, Arlington, Texas, United States of America; 3 Factor-Inwentash Faculty of Social Work, University of Toronto, Toronto, Ontario, Canada; 4 Institute for Life Course and Aging, University of Toronto, Toronto, Canada; 5 Department of Family and Community Medicine, Temerty Faculty of Medicine, University of Toronto, Toronto, Ontario, Canada; 6 Bloomberg Faculty of Nursing, University of Toronto, Toronto, Ontario, Canada; Chinese Academy of Medical Sciences and Peking Union Medical College, CHINA

## Abstract

Although studies have investigated the association between adverse childhood experiences and chronic health outcomes including stroke, few studies have investigated the association between parental divorce and stroke among adults with no history of childhood abuse. The objectives of this study were to investigate the association between parental divorce in childhood and stroke in older adulthood among those who did not experience child abuse and to examine whether this association differs between men and women. This study utilized population-based data from the 2022 Behavioral Risk Factor Surveillance System. An analytic sample of 13,205 adults aged 65 and above (56.6% female) who have never experienced childhood physical nor sexual abuse were analyzed using binary logistic regression. The outcome variable investigated was self-report of a physician-diagnosis of stroke, and the main exposure of interest was parental divorce. In this sample of older adults, 7.3% reported having stroke, while 13.9% reported that their parents had divorced before the respondent was 18 years old. Controlling for the effects of other factors, respondents who experienced parental divorce had 1.61 times higher odds of having a stroke when compared to their counterparts who did not experience parental divorce (AOR = 1.61, 95% CI = 1.15–2.24). The association between parental divorce and stroke was not dependent on sex; however, compared to females, males had 1.47 times higher odds of having a stroke (AOR = 1.47, 95% CI = 1.11–1.93). The findings of this study suggest that individuals in this cohort whose parents divorced as children were at greater risk for stroke later in life. Potentially moderating variables were hypothesized, including childhood poverty, sleep hygiene, and hypertension.

## Introduction

Each year approximately 795,000 individuals in the United States (U.S.) have a stroke [1, 2]. For one in five of these individuals, the stroke is fatal, making strokes the fifth leading cause of death in the U.S. [3]. An estimated two-thirds of stroke survivors experience impaired mobility [4], over half of survivors experience cognitive impairment [1], and many can no longer live independently in the community [5]. In addition to the substantial direct economic impact of medical care costs, there are many indirect consequences of a stroke, including lost productivity and increased care needs [6]. The combined direct and indirect annual cost of stroke to the U.S. economy is estimated to be about $56.5 billion [7].

There are many known risk factors for stroke. Sociodemographic risk factors for stroke include older age [7], female sex [7], being Black [8], being widowed or divorced [9], lower socioeconomic status (SES) [10], and living in rural areas [11]. Health behaviors that increase the risk of stroke include smoking, heavy drinking, inadequate physical activity, and higher body mass index (BMI) [12, 13]. Given the high prevalence of stroke and the significant economic burden associated with the disease, it is important to gain more knowledge about potentially modifiable risk factors.

Recent research suggests that adverse childhood experiences (ACEs) increase the risk of stroke in adulthood [14, 15]. ACEs are a broad term that encompasses distressing and potentially harmful events of sexual, physical or emotional abuse, neglect, or family dysfunction such as parental incarceration, parental mental illness, parental substance abuse, or parental divorce [16–18]. Estimates shows that more than three in five American adults experienced at least one ACE in their childhood, with one in six experiencing four or more ACEs [16, 18]. The number of ACEs experienced increases the risk for negative health outcomes, including having a stroke [14, 15, 19]. Research indicates that women who experienced sexual or physical abuse as a child are at greater risk for stroke [20] and both men and women with a history of childhood maltreatment are at higher risk for cardiovascular disease [21, 22]. Exposure to physical neglect as a child also increases stroke risk [23].

Although studies have investigated the association between ACEs and chronic health outcomes including stroke, few studies have investigated the association between parental divorce and stroke among adults with no history of childhood abuse. Parental divorce or separation has been found to be associated with a range of adverse health outcomes in adulthood including poorer mental health [24–26], suicidal ideation [27], lower self-rated health [28], and higher lifetime morbidity [29]. Parental divorce has also been linked with unhealthy behaviors and conditions such as cigarette smoking [30, 31], substance use disorders [32], and obesity [33]. In turn, these health risk behaviors increase the risk of stroke [12, 34–39]. Other known risk factors for stroke such as depression [40] and diabetes [41], are also elevated among adults who experienced parental separation or divorce during childhood [26, 29].

Our previous research examined the association between parental divorce and stroke using the population-based 2010 Behavioral Risk Factor Surveillance System [42]. Because our interest was in parental divorce in the absence of other childhood adversities, our analysis excluded those who had, during their childhood, experienced parental substance abuse, parental domestic violence, or any form of childhood abuse (physical, sexual, or emotional). Even after accounting for sociodemographic factors, our study found that parental divorce significantly elevated the odds of stroke among men aged 18 and older [42]. Although the odds of stroke were elevated among women who had experienced parental divorce, this association did not reach statistical significance. However, the number of women with a stroke in our previous study was relatively small (n = 129) and the age range was 18 and older [42], despite the majority of strokes occurring in later life [7]. To our knowledge, this study has not yet been replicated with more recent population-based data or with a focus solely on stroke as opposed to a combination of stroke and heart disease (e.g. [43]).

Thus, using a large representative sample of community-dwelling Americans aged 65 and older, the objectives of this study were: (1) To investigate the cross-sectional association between parental divorce in childhood and stroke in older adulthood among those who did not experience child abuse; and (2) To examine whether this association differs between men and women. We hypothesized that, controlling for sociodemographic characteristics, other ACEs, social support, health behaviors, chronic health conditions, and the presence of an adult who made the respondent feel safe and protected during childhood, respondents who experienced parental divorce during childhood would have higher odds of having a stroke. We further hypothesized that the association between parental divorce and stroke vary by sex.

## Materials and methods

### Data source and sample

Our study was based upon secondary analysis of the public use dataset of the Behavioral Risk Factor Surveillance System (BRFSS) [44] funded by the Centers for Disease Control and Prevention (CDC). Because the public-use data have no individually identifiable information, ethics approval was not required to conduct the current study. The data was accessed for research purposes on July 16, 2024. The BRFSS is a cross-sectional survey conducted annually by the CDC to gather data on health-related risk behaviors, chronic health conditions, and use of preventive services from non-institutionalized U.S. adult population aged 18 and above [45]. Detailed information on the design of the 2022 BRFSS, including the objectives, methodology, and sampling procedure, are available from the U.S. Department of Health and Human Services and the CDC [46]. The authors have also provided a detailed description of the methods and variables in other publications [31, 42]. The 2022 BRFSS was approved by the CDC’s Institutional Review Board, and the de-identified data are publicly available. The BRFSS questionnaire consists of core components, optional modules, and state-added questions. The 2022 optional survey module on ACEs was answered by all respondents aged 18 and older in eight states (Arkansas, Florida, Iowa, Nevada, North Dakota, Oregon, South Dakota, and Virginia). The initial sample size for the 2022 BRFSS was 445,132 respondents across all 50 states. When the sample was restricted to individuals aged 65 and older who lived in the eight states that administered the ACEs module and who had not been sexually or physically abuse, the sample size decreased to 14,454. Of these 14,454 respondents, a total of 1,249 individuals (8.6%) had missing data on one or more of the variables included in the analysis, and these respondents were excluded using listwise deletion, resulting in a final sample size was 13,205. The prevalence of missing data was only 0.3% for the outcome of interest (stroke), and 1.0% for the key exposure of interest (parental divorce). The prevalence of missing data among the control variables ranged from a high of 1.5% (i.e., heavy alcohol use) to a low of 0.2% (i.e., level of education). Research suggests that using listwise deletion when less than 10% of the data are missing provides comparable estimates to other methods of dealing with missing data [47].

### Outcome variable

The outcome variable examined in this study was self-reported physician diagnosis of stroke, and it was measured as a binary variable based on response to the question, “Has a doctor, nurse, or other health professional EVER told you that you had a stroke?” Respondents who answered “Yes” were coded as 1, whereas respondents who answered “No” were coded as 0. This item has been used in other population-based studies and provides reliable overall measure of stroke [42, 48, 49].

### Main exposure of interest

The main exposure of interest examined in this this study was parental divorce and was measured as a binary variable based on response to the question, “Before you were 18 years of age, were your parents separated or divorced?” with the following response options, “Yes,” “No,” and “Parents not married.” Respondents who answered “Yes” were coded as 1, whereas respondents who answered “No” were coded as 0. Respondents whose parents were not married were excluded from the analysis.

### Covariates

Covariates examined in this study were grouped under SES (education and household income), ACEs (emotional abuse, household mental illness, household substance use, household incarceration, and witnessed domestic violence), feeling safe and protected by an adult in the household, social support (marital status and social and emotional support), health behaviors (heavy drinking, cigarette smoking, BMI, and physical activity), and health conditions (depression and diabetes). Detailed information about how each covariate was measured and the exact questionnaire wording is provided in S1 Table (See S1 Table).

### Demographic characteristics

The following demographic characteristics were considered. Age was measured as an ordinal variable into the following categories “0 = 65–69 years,” “1 = 70–74 years,” “2 = 75–79 years,” and “3 = 80 years and above.” Sex of the respondent was coded as a binary variable with female as the reference category. Race/ethnicity was measured as a nominal variable into the following categories “Non-Hispanic White,” “Non-Hispanic Black,” and “Hispanic, Other race.” Rural/urban status was coded as a binary variable with urban as the reference category.

### Data analyses

The analytic procedure involved using descriptive, bivariate, and multivariable analytical techniques. First, the general distribution of all the variables was examined using frequencies and percentages. Second, Pearson chi-square test of association was conducted to examine the bivariate association between the study variables and stroke. Third, binary logistic regression was then employed to examine the cross-sectional association between parental divorce and stroke among respondents who have never experienced childhood physical or sexual abuse. Three logistic regression models were fitted. In Model 1, we regressed stroke on parental divorce and the demographic variables given their a priori importance. Model 2 consists of variables in Model 1 plus SES factors (education and household income). Model 3, which is the fully adjusted model, consists of variables in Model 2 plus all the other covariates. To investigate whether the effects of parental divorce on stroke is dependent on sex, we conducted a two-way interaction between parental divorce and sex on stroke while simultaneously controlling for the effects of demographic characteristics and other covariates. We found that the interaction was not significant, hence we reverted to examining the main effects. Adjusted odds ratio (AOR) and 95% Confidence Intervals (CI) are reported. All analyses were two-tailed, and variables were considered significant if the *p*-value was less than .05 or the 95% CI does not contain 1. To account for the weighting and complexity of the sampling design employed by the BRFSS, we used Stata’s “svyset” command. Although missing data were handled using listwise deletion, sampling weights were still applied to the remaining data to ensure that the analysisrepresents the population proportions as intended in the original sampling design. This is especially important in complex survey data such as the BRFSS where the sample is not representative of the population without weighting. All analyses were performed using Stata version MP 17.

## Results

### Sample characteristics

Table 1 shows the general distribution of the sample characteristics and the bivariate association between stroke and the sample characteristics. Of the 13,205 adults aged 65 and above who have never experienced childhood physical or sexual abuse, 7.3% had self-reported a physician diagnosis of stroke. About one in seven respondents (13.9%) experienced parental divorce. There were significant bivariate associations between a number of variables and stroke. For instance, 11.2% of respondents who experienced parental divorce, compared to 7.5% of respondents who did not experience parental divorce, had a stroke (χ^2^(1) = 24.70, *p* < .001). Respondents were more likely to have a stroke if they: were older, were male, lived in a rural county, had low income, experienced neglect, experienced household substance use, witnessed domestic violence, or did not feel safe and protected by an adult in the household all the time. About 10.7% of respondents who were separated/divorced compared to 8.8% of respondents who were widowed, 7.9% of respondents who were single/never married, and 6.7% of respondents who were married reported that they had been diagnosed with a stroke (χ^2^(3) = 37.40, *p* < .001). The prevalence of stroke was significantly higher amongrespondents who currently smoked (11.2%) compared to former smokers (8.5%) and those who had never smoked (7.1%) (χ^2^(2) = 24.51, *p* < .001). About 10.9% of respondents who were physically inactive compared to 6.7% of respondents who were physically active reported that they had been diagnosed with a stroke (χ^2^(1) = 68.45, *p* < .001). The prevalence of stroke was significantly higher among respondents diagnosed with depression (11.2%) compared to those without a diagnosis of depression (7.4%; χ^2^(1) = 27.25, *p* < .001). Similarly, the prevalence of stroke was significantly higher among respondents diagnosed with diabetes (11.9%) compared to those without a diagnosis of diabetes (6.8%; χ^2^(1) = 78.53, *p* < .001).

**Table 1:** Sample characteristics and percent with diagnosis of stroke (N = 13,205).

Variable			Unweighted Frequency	Weighted %	% with Stroke	Chi-square (p-value)
**Outcome variable**						
	Stroke					
		No	12,164	92.7		
		Yes	1,041	7.3		
**Exposure variable of interest**						
	Parental divorce					
		No	11,781	86.1	7.5	24.70 (*p* < .001)
		Yes	1,424	13.9	11.2	
**Demographic variables**						
	Age					69.58 (*p* < .001)
		65–69 years	3,572	27.5	5.9	
		70–74 years	3,516	30.2	6.7	
		75–79 years	2,773	19.1	8.4	
		80 years and above	3,344	23.2	10.9	
	Sex					17.65 (*p* < .001)
		Female	7,627	56.6	7.0	
		Male	5,578	43.4	9.0	
	Race					5.30 (*p* = .070)
		Non-Hispanic White	12,015	79.1	7.8	
		Non-Hispanic Black	588	8.5	10.4	
		Hispanic/Other race	602	12.4	7.5	
	Rural urban county					5.90 (*p* = .015)
		Urban county	10,278	90.3	7.6	
		Rural county	2,927	9.7	9.0	
**Socioeconomic status**						
	Education					63.55 (*p* < .001)
		Graduated from college/technical school	5,318	30.8	6.0	
		Attended college/technical school	3,820	31.8	7.7	
		High school or less	4,067	37.4	10.5	
	Household income					123.56 (*p* < .001)
		$75,000 and above	3,192	24.2	5.3	
		$50,000-$74,999	2,097	13.7	7.2	
		$35,000-$49,999	1,932	12.0	7.3	
		$20,000-$34,999	2,318	17.6	10.3	
		Less than $20,000	864	8.0	15.6	
		Missing data	2,802	24.5	7.4	
**Adverse childhood experiences**						
	Emotional abuse					0.50 (*p* = .477)
		No	11,100	85.0	7.8	
		Yes	2,105	15.0	8.3	
	Neglect					17.68 (*p* < .001)
		No	12,959	96.1	7.8	
		Yes	246	3.9	15.0	
	Household mental illness					0.63 (*p* = .426)
		No	12,307	94.2	7.8	
		Yes	898	5.8	8.6	
	Household substance use					9.76 (*p* = .002)
		No	11,034	85.2	7.6	
		Yes	2,171	14.8	9.5	
	Household incarceration					3.50 (*p* = .061)
		No	12,972	97.8	7.8	
		Yes	233	2.2	11.2	
	Witnessed domestic violence					4.32 (*p* = .038)
		No	12,444	94.1	7.8	
		Yes	761	5.9	9.9	
	Felt safe and protected by an adult in the household all the time					4.96 (*p* = .026)
		Yes	11,293	84.4	7.7	
		No	1,912	15.6	9.2	
**Social support**						
	Marital status					37.40 (*p* < .001)
		Married	6,996	59.1	6.7	
		Separated/Divorced	1,714	12.0	10.7	
		Widowed	3,636	23.4	8.8	
		Single/Never married	859	5.5	7.9	
	Social and emotional support					7.12 (*p* = .130)
		Always	3,222	35.9	7.9	
		Usually	1,629	15.9	6.9	
		Sometimes	475	5.3	7.8	
		Rarely/never	237	3.2	11.8	
		Missing data	7,642	39.7	8.0	
**Health risk behaviors**						
	Heavy alcohol use					0.71 (*p* = .401)
		No	12,590	95.2	7.9	
		Yes	615	4.8	7.0	
	Cigarette smoking					24.51 (*p* < .001)
		Never	7,451	56.4	7.1	
		Formerly smoked	4,745	36.4	8.5	
		Currently smoke	1,009	7.2	11.2	
	BMI					19.04 (*p* < .001)
		Normal	4,005	30.8	7.5	
		Overweight	4,740	34.9	8.0	
		Obese	3,811	29.0	8.8	
		Missing	649	5.3	4.0	
	Physical activity					68.45 (*p* < .001)
		Inactive	3,805	29.5	10.9	
		Active	9,400	70.5	6.7	
**Chronic health conditions**						
	Depression					27.25 (*p* < .001)
		No	11,593	87.6	7.4	
		Yes	1,612	12.4	11.2	
	Diabetes					78.53 (*p* < .001)
		No	10,436	76.7	6.8	
		Yes	2,769	23.3	11.9	

### Multivariable logistic regression results

Table 2 shows the results of the multivariable logistic regression analysis of the association between parental divorce and stroke among respondents who have never experienced childhood physical or sexual abuse. Controlling for demographic factors in Model 1, we found that respondents who experienced parental divorce had 1.73 times higher odds of having a stroke when compared to their counterparts who did not experience parental divorce (AOR = 1.73, *p* = .001, 95% CI = 1.26–2.37). This significant association was partially attenuated with the addition of SES factors in Model 2, and other covariates in Model 3. In the fully adjusted model, respondents who experienced parental divorce had 1.61 times higher odds of having a stroke when compared to their counterparts who did not experience parental divorce (AOR = 1.61, *p* = .005, 95% CI = 1.15–2.24).

**Table 2:** Multivariable logistic regression analysis of the association between parental divorce and stroke^a^ (N = 13,205).

Variables			Model 1		Model 2		Model 3	
			AOR (95% C.I)	*p*-value	AOR (95% C.I)	*p*-value	AOR (95% C.I)	*p*-value
**Key variable of interest**								
	Parental divorce (No)							
		Yes	1.73 (1.26–2.37)	.001	1.65 (1.20–2.28)	.002	1.61 (1.15–2.24)	.005
**Demographic variables**								
	Age (65–69 years)							
		70–74 years	0.92 (0.63–1.33)	.654	0.91 (0.63–1.31)	.611	0.93 (0.65–1.33)	.699
		75–79 years	1.72 (1.16–2.54)	.007	1.68 (1.14–2.49)	.009	1.74 (1.17–2.57)	.006
		80 years and above	2.09 (1.43–3.03)	< .001	1.89 (1.29–2.79)	.001	2.11 (1.38–3.21)	.001
	Sex (Female)							
		Male	1.36 (1.05–1.76)	.019	1.41 (1.08–1.83)	.010	1.47 (1.11–1.93)	.006
	Race (Non-Hispanic White)							
		Non-Hispanic Black	1.16 (0.75–1.79)	.515	1.02 (0.64–1.62)	.933	0.98 (0.62–1.53)	.921
		Hispanic/Other race	0.28 (0.14–0.57)	< .001	0.22 (0.11–0.46)	< .001	0.22 (0.11–0.47)	< .001
	Rural urban county (Urban county)							
		Rural county	1.29 (1.00–1.68)	.050	1.10 (0.84–1.45)	.498	1.06 (0.82–1.38)	.639
**Socioeconomic status**								
	Education (Graduated from college/technical school)				1.12 (0.83–1.49)	.458	1.09 (0.81–1.47)	.569
		Attended college/technical school			1.39 (1.05–1.85)	.020	1.35 (1.02–1.78)	.037
		High school or less						
	Household income ($75,000 and above)							
		$50,000-$74,999			1.55 (1.07–2.26)	.021	1.42 (0.98–2.07)	.067
		$35,000-$49,999			1.50 (1.00–2.26)	.053	1.35 (0.88–2.06)	.169
		$20,000-$34,999			2.50 (1.70–3.66)	< .001	2.13 (1.45–3.11)	< .001
		Less than $20,000			2.60 (1.60–4.21)	< .001	2.01 (1.23–3.29)	.006
		Missing data			1.29 (0.89–1.89)	.182	1.18 (0.77–1.80)	.441
**Adverse childhood experiences**								
	Emotional abuse (No)							
		Yes					1.10 (0.79–1.53)	.585
	Neglect (No)							
		Yes					0.93 (0.42–2.07)	.858
	Household mental illness (No)							
		Yes					1.03 (0.69–1.54)	.886
	Household substance use (No)							
		Yes					1.11 (0.82–1.50)	.514
	Household incarceration (No)							
		Yes					0.89 (0.44–1.81)	.755
	Exposure to domestic violence (No)							
		Yes					1.09 (0.63–1.87)	.756
	Felt safe and protected all the time (Yes)							
		No					0.87 (0.62–1.23)	.447
**Social support**								
	Marital status (Married)							
		Divorced/separated					1.38 (0.99–1.92)	.056
		Widowed					0.96 (0.67–1.37)	.802
		Single/Never married					0.99 (0.63–1.54)	.948
	Emotional support (Always)							
		Usually					0.81 (0.51–1.27)	.352
		Sometimes					0.71 (0.38–1.35)	.302
		Rarely/never					1.23 (0.62–2.43)	.550
		Missing data					1.09 (0.80–1.50)	.574
**Health risk behaviors**								
	Heavy alcohol use							
		Yes					0.82 (0.48–1.39)	.459
	Cigarette smoking (Never)							
		Formerly smoked					1.03 (0.79–1.35)	.814
		Currently smoke					1.17 (0.78–1.74)	.442
	BMI (Normal)							
		Overweight					0.93 (0.69–1.27)	.659
		Obese					0.95 (0.70–1.28)	.725
		Missing					1.10(0.43–0.83)	.843
	Physical activity (Inactive)							
		Active					0.75 (0.58–0.98)	.035
**Chronic health conditions**								
	Depression							
		Yes					1.76 (1.22–2.54)	.002
	Diabetes (No)							
		Yes					1.37 (1.03–1.81)	.031

Reference category is indicated in parenthesis

^a^Among respondents who had never experienced childhood physical or sexual abuse

Compared to respondents aged 65–69, the odds of having a stroke were 1.74 times higher for respondents aged 75–79 (AOR = 1.74, *p* = .006, 95% CI = 1.17–2.57) and 2.11 times higher for respondents aged 80 and above (AOR = 2.11, *p* < .001, 95% CI = 1.38–3.21). Compared to females, males had 1.47 times higher odds of having a stroke (AOR = 1.47, *p* = .006, 95% CI = 1.11–1.93). Respondents were more likely to have a stroke if they make less than $35,000 in household income. Depression and diabetes were both significantly positively associated with stroke. Respondents diagnosed with depression had 1.76 times higher odds of having a stroke (AOR = 1.76, *p* = .002, 95% CI = 1.22–2.54) and respondents diagnosed with diabetes had 1.37 times higher odds of having a stroke (AOR = 1.37, *p* = .031, 95% CI = 1.03–1.81) both when compared to their counterparts without such diagnoses.

## Discussion

The objectives of this study were to examine the association between parental divorce and stroke, and whether this association differs between men and women using a large, representative, cross-sectional sample of community-dwelling older Americans with no history of childhood physical and/or sexual abuse. When compared with those who did not experience parental divorce, children of divorced parents had 1.61 times higher odds of having a stroke. Our current study found that the interaction between divorce and sex on stroke was statistically not significant. That is to say, the association between parental divorce and stoke did not vary by sex. However, there was a significant main effect of sex on stroke with males having 1.47 times higher odds of having a stroke when compared to females.

The non-significant interaction between divorce and sex on stroke in our current study is in contrast to our previous study in which the parental divorce -stroke association was only significant for men (AOR = 3.01; 95% CI = 1.68, 5.39), albeit with very wide confidence intervals. Although the association between parental divorce and stroke for women in our earlier study generated odds very similar (AOR = 1.64; 95% CI = 0.89, 3.02) to the ones for the combined sexes in our current study (AOR = 1.61, 95% CI = 1.15–2.24), the association in our earlier study did not reach statistical significance for women [42]. The current study is focused on a higher risk subgroup (those aged 65 and older vs. those aged 18 and older). We therefore found a higher percentage of women who reported a stroke in our current study (7.3%) versus 2.13% in our previous study [42], which leads to greater statistical power. We anticipate that the presence of a statistically significant association between parental divorce and stroke for women in the current study but not in our earlier study could be due to the greater statistical power in the current study.

The association between parental divorce and stroke in the current study remained even after controlling for known risk factors of stroke, such as diabetes [41], depression [40], and a small social support network [50]. It is interesting to note that the association between parental divorce and stroke is comparable in magnitude to the association between two well established risk factors for stroke: diabetes and depression. Respondents with diabetes had 1.37 times higher odds of having a stroke when compared to those without diabetes, respondents with depression had 1.76 times higher odds when compared to those without depression, and respondents with a history of parental divorce had 1.61 times higher odds when compared to those without a history of parental divorce. The connections between depression and stroke [40, 51, 52] and diabetes and stroke [53–55] have been significant areas of focus in the research literature. In contrast, the connection between parental divorce and stroke risk remains understudied; to our knowledge, no research study with a sole focus on parental divorce and stroke as opposed to a combination of stroke and heart disease (e.g., [43]) has been conducted since the publication of our previous study [42]. By specifically focusing on stroke in our research, we were able to isolate and measure a distinct acute cardiovascular event and examine the factors associated with it.

Childhood physical and sexual abuse have been found to be significantly associated with adult stroke risk [20]. Finding an association between parental divorce and stroke risk in the absence of childhood sexual and physical abuse is an important addition to the extant literature suggesting that even when a child did not experience physical or sexual abuse, their parents’ marriage dissolution may be associated with adverse long-term health outcomes including stroke.

Although we cannot determine the mechanism linking parental divorce and stroke with our current data, we have several hypotheses which warrant future research. Parental divorce is a source of substantial stress for many children, as displayed through higher rates of emotional and behavioral disruption [56] and poorer mental health [57] following parental divorce. The Biological Embedding theory presents an explanation as to how exposure to prolonged stress could lead to long-term negative health outcomes [58]. This theory purports that exposure to high levels of stress during childhood may disrupt the hypothalamic-pituitary-adrenal (HPA) axis, the brain’s stress response pathway [58]. In response to stress, the HPA axis stimulates the release of hormones which lead to the adrenal glands releasing glucocorticoids, primarily cortisol [59]. Working in tandem with the autonomic nervous system and immune systems, the HPA axis prepares the body to respond to stressful situations [60]. When a child is exposed to prolonged stress, such as the stress associated with parental divorce (parental fighting, household tension, moving to a new home or school, etc.), their HPA axis may become dysregulated [61]. Previous research has demonstrated that disruption to the HPA axis is associated with cardiovascular disease [62] and major depressive disorder [63]. Thus, it is possible that parental divorce may act as a catalyst for chronic stress which has the potential to disrupt the HPA axis, which in turn could heighten stroke risk in adulthood. Because of the use of a cross-sectional dataset and the fact that the BRFSS did not include information on HPA axis, our current study cannot confirm that HPA axis dysregulation is the mechanism connecting parental divorce and stroke risk. Future researchers should consider using longitudinal designs in order to better study this connection. However, the existing research literature does suggest that stress increases the risk of stroke [64] and dysregulation to the HPA axis is associated with worse stroke outcomes [65, 66].

Previous research has also indicated a link between hypertension (high blood pressure) and both parental divorce [67] and stroke risk [13]. Children of divorced parents are at risk for developing hypertension [67], and individuals with hypertension are at elevated risk for stroke [13]. The dataset used for our current study did not include information on hypertension; thus, we were unable to examine whether hypertension mediated the association between parental divorce and stroke risk in our study. Future research should examine whether life-time hypertension mediates the association between parental divorce and stroke risk.

Furthermore, previous research has established that individuals who experience sleep disorders are at greater risk for stroke [68]. Parental divorce has also been found to be associated with sleep disruption among children [69] with the negative effects of parental divorce on insomnia symptoms persisting into adulthood [70]. The BRFSS dataset did not include information on sleep, and as a result, we were unable to control for sleep disorders. Future research should examine whether sleep disorders or sleep disruption play a role in mediating the association between parental divorce and stroke.

Another factor that may help explain the association between parental divorce and stroke risk is childhood poverty. In the current study, we controlled for respondents’ current income, but did not have information on respondents’ income during childhood. There is a higher prevalence of parental divorce among low-income families [71, 72] and an increased risk of poverty particularly for women and children following a divorce [73–76]. The extant literature has found that children living in poverty are at greater risk for negative health outcomes including obesity [77] and chronic stress [78]. In addition, childhood poverty has been found to be a significant social determinant of health [79], and an important risk factor for stroke in adulthood [80–83]. Given the link between childhood poverty and stress [78], and between stress and stroke [64],future studies should take into account measures of childhood poverty to uncover the role it my play in the association between parental divorce and stroke.

In keeping with existing research literature, this study also found the following factors were associated with increased odds of stroke: having diabetes [41], having major depressive disorder [40], being divorced or widowed [9], older age [7], being Black [8], having low socioeconomic status [10], having low physical activity [13], and smoking [36]. The association found between sex and stroke risk is worth noting. Our findings showed that compared to females, males had 1.47 times higher odds of having a stroke. This finding contradicts past research that has found that compared to men, women have a higher life-time incidence of stroke [7]. Our cohort was 65+ and previous research has found that during this period of late adulthood, women have a one in five risk of stroke while men have approximately one in six risk of stroke [7].

Another finding worth noting is that a number of ACEs (i.e. neglect, witnessing domestic violence, not feeling safe and protected by an adult at home, and parental substance use) were significantly associated with stroke at the bivariate level, but once we controlled for other factors, these associations became non-significant in the multivariable logistic regression models. Previous research has established that those who experienced a greater number of ACEs ihave a higher stroke risk; however, those studies included individuals who had been physically and/or sexually maltreated in childhood [15], and our study did not.

### Limitations

Our study has a number of limitations that should be discussed. The first limitation is related to the survivor selection effect. The respondents in this study, by nature of having survived past 65, may not be representative of individuals who had strokes earlier in life which resulted in premature mortality or left them too incapacitated to complete a survey such as the BRFSS. Longevity and health are influenced by a number of biological and environmental factors [84] including greater social support [85], increased religious practice [86], and fewer unhealthy behaviors (e.g., smoking and excessive alcohol consumption) [84]. Thus, we anticipate that our sample of older Americans would have higher general well-being and have had fewer childhood adversities than those in their birth cohort who died prematurely. However, such a selection effect would have biased the results of the study towards the null, making it more difficult to establish a significant association between parental divorce and stroke.

Another factor worth noting and a potential limitation to the generalizability of these findings to future cohorts of older adults is that rates of parental divorce were quite rare in this cohort; just over 10% experienced parental divorce. The youngest of this cohort were born in 1957, over a decade before the U.S.’s first no-fault divorce bill was signed in 1969 [87]. Rates of divorce changed significantly over the second half of the 21^st^ century: 2.2 per 1,000 population in 1959, 3.2 in 1969, 5.3 in 1979, 4.7 in 1989, and 4.2 in 1999 [88, 89]. The cohort examined in this study experienced parental divorce just before a national increase in divorce. We anticipate that our cohort experienced higher levels of divorce-related stigma from their community and peers during their childhood than did those in later birth cohorts, who grew up when parental divorce would have been more normative. Furthermore, among those in the cohort whose parents divorced, it may be that the level of parental conflict was particularly high in order that the parents would instigate a divorce in a time when divorce was more stigmatized and more challenging to obtain than it was in later years.

Another limitation is that we do not know at what point during their childhood the participants experienced parental divorce, nor the level of contact they had with the non-custodial parent. Previous research indicates that the impact of divorce varies by the child’s age [90] and by the level of involvement of the non-custodial parent post-divorce [91]. Future research should focus on whether the age a child experiences parental divorce and the level of involvement with the non-custodial parent moderates future stroke risk. A limitation to the validity of this study was the reliance on self-report of a medical diagnosis of stroke which could allow for participant recall or reporting biases. It would have been preferable to have a chart-review. However, the sensitivity and specificity of self-report of a medical diagnosis of stroke has been found to be very high (95%) [92] and the item used in measuring stroke in this study has been used in other population-based studies and has been found to provide a reliable overall measure of stroke [48, 49]. Another limitation was the lack of information in the dataset on stroke characteristics, such as type of stroke (ischemic, hemorrhagic, etc.), severity of stroke, and age at which it occurred. Research supports that some factors elevate the risk of a particular type of stroke over others; for example, hypertension is more strongly associated with risk of intracerebral hemorrhage than ischemic stroke [13]. Thus, future research should examine whether parental divorce is associated with greater risk for a particular type of stroke. If this is the case, then this may help illuminate the mechanism by which parental divorce and stroke risk are connected.

Moreover, we were not able to control for several known risk factors for stroke including genetic predisposition [93], lifetime use of oral contraceptives [94], history of transient ischemic attacks [95], magnitude and duration of high blood pressure [13], high blood cholesterol [96], or high red blood cell counts [97], as these measures were not available in the BRFSS. Future research should examine the potential that these factors have to mediate the relationship between parental divorce and stroke risk.

Lastly, our use of a cross-sectional design limits interpretation of the study’s findings. Although we found evidence that experiencing parental divorce is associated with higher stroke risk in adulthood, we cannot interpret this association to be causal. Future research should employ longitudinal designs, rather than rely on retrospective recall of early childhood experiences such as parental divorce, in order to better examine the potential for a causal relationship between parental divorce and stroke incidence.

## Conclusion

In conclusion, we found that experiencing parental divorce in childhood was associated with increased odds of stroke in a population-based cohort of Americans aged 65 and older who had never experienced childhood physical or sexual abuse. The findings built on our earlier study on the topic [42] in a few key ways. Our previous study used data from the 2010 BRFSS while our current study uses data from the 2022 BRFSS survey. This means that our current study is examining the next generation of older Americans. Before analyzing this newer data set, it was unclear if, because of societal and cultural changes between and during the World Wars, the connection found between parental divorce and stroke risk in the older cohort would be found again in a more recent cohort of older Americans. Thus, replicating these earlier findings with a newer cohort contributed to our knowledge of the association. Our previous study found support for a significant association between parental divorce and stroke risk, but only among men and not among women. This current study used a much larger sample of older women and men, providing us with greater statistical power. Our findings advance the body of knowledge by demonstrating that both men and women aged 65 and older in this more recent cohort were significantly at greater risk of stroke when they had a history of parental divorce. We found that the association between parental divorce and stroke was of similar magnitude to two well-established risk factors for stroke: diabetes [41] and major depressive disorder [40]. Possible explanations were discussed, such as biological embedding and the impact of childhood poverty. We discussed how the cohort in question experienced parental divorce before a national increase in the prevalence of divorce and, thus, it is important to consider how other contextual factors, such as community stigma related to divorce and high familial conflict, which may have affected the cohort’s health and well-being. Due to the changes in societal norms, it is not clear that Gen X or Millennial Americans will experience a similar link between parental divorce and stroke as was evident in our sample from the Baby Boom and Silent Generation cohorts. Future research is needed to investigate generational differences in the parental divorce-stroke association. No association was found between childhood emotional abuse, parental domestic violence, parental incarceration, parental mental illness, or parental substance use and stroke risk, once a wide range of sociodemographic factors were taken into account. The following factors were found to be associated with stroke incidence in this sample: higher age, non-Hispanic Black ethnicity, male sex, and lower socioeconomic status (measured through education level and household income). Because the association between parental divorce and stroke remained even after controlling for many common risk factors, future research is needed to clarify the potential pathways causing this association.

## Supporting information

S1 Table List of covariates. (DOCX)
