# Supplementary material for: Parental divorce’s long shadow: Elevated stroke risk among older Americans
Source: PLoS One. 2025 Jan 22;20(1):e0316580. doi: 10.1371/journal.pone.0316580 (PMC11753648; doi:10.1371/journal.pone.0316580)
Supplement: S1 Table — (DOCX) [file pone.0316580.s001.docx]

**S1 Appendix**

**Supplementary Table S1**

List of covariates

| Variable name | | Survey question(s) | Analytic coding |
| --- | --- | --- | --- |
| **Socioeconomic status** | |  |  |
|  | Highest level of education | What is the highest grade or year of school you completed? | 0 = High school or less  1 = Some college  2 = College degree or higher |
|  | Income | Is your annual household income from all sources? | 0 = Less than $25,000  1 = $25,000-$34,999  2 = $35,000-$49,999  3 = $50,000-$99,999  4 = $100,000 and above  5 = Missing |
| **Adverse childhood experiences** | |  |  |
|  | Emotional abuse | How often did a parent or adult in your home ever swear at you, insult you, or put you down? | 0 = Never  1 = Once/More than once |
|  | Neglect | For how much of your childhood was there an adult in your household who tried hard to make sure your basic needs were met? Would you say never, a little of the time, some of the time, most of the time, or all of the time? | 0 = Most/all of the time  1 = Never/a little of the time/some of the time |
|  | Household mental illness | Did you live with anyone who was depressed, mentally ill, or suicidal? | 0 = No  1 = Yes |
|  | Household substance use | Did you live with anyone who was a problem drinker or alcoholic? | 0 = No  1 = Yes |
|  |  | Did you live with anyone who used illegal street drugs or who abused prescription medications? |  |
|  | Household incarceration | Did you live with anyone who served time or was sentenced to serve time in a prison, jail, or other correctional facility? | 0 = No  1 = Yes |
|  | Witnessed domestic violence | How often did your parents or adults in your home ever slap, hit, kick, punch or beat each other up | 0 = Never  1 = Once/More than once |
|  | Felt safe and protected | For how much of your childhood was there an adult in your household who made you feel safe and protected? Would you say never, a little of the time, some of the time, most of the time, or all of the time? | 0 = All of the time  1 = Not all of the time |
| **Social support** | |  |  |
|  | Marital status | Are you (marital status)? | 0 = Married  1 = Divorced/Separated  2 = Widowed  3 = Single/Never married |
|  | Social and emotional support | How often do you get the social and emotional support you need? | 0 = Always  1 = Usually  2 = Sometimes  3 = Rarely/Never  4 = Missing |
| **Health risk behaviors** | |  |  |
|  | Heavy drinking | Heavy drinkers (adult men having more than 14 drinks per week and adult women having more than 7 drinks per week) | 0 = No  1 = Yes |
|  | Smoking status | Have you smoked at least 100 cigarettes in your entire life? Do you now smoke cigarettes every day, some days, or not at all? | 0 = Never  1 = Former  2 = Current |
|  | BMI category | Three-categories of Body Mass Index (BMI) | 0 = Normal BMI  1 = Overweight  2 = Obese  3 = Missing |
|  | Physically active | During the past month, other than your regular job, did you participate in any physical activities or exercises such as running, calisthenics, golf, gardening, or walking for exercise? | 0 = No  1 = Yes |
| Chronic health condition | |  |  |
|  | Depression | Has a doctor, nurse, or other health professional EVER told you that you had a depressive disorder (including depression, major depression, dysthymia, or minor depression)? | 0 = No  1 = Yes |
|  | Diabetes | Has a doctor, nurse, or other health professional EVER told you that you had diabetes? | 0 = No  1 = Yes |
